# Shedding light on conditions for the successful passive dissemination of recommendations in primary care: a mixed methods study

**DOI:** 10.1186/s13012-018-0822-x

**Published:** 2018-10-16

**Authors:** Isabelle Vedel, Melanie Le Berre, Nadia Sourial, Geneviève Arsenault-Lapierre, Howard Bergman, Liette Lapointe

**Affiliations:** 1 Lady Davis Institute of Medical Research, Jewish General Hospital, 3755 Côte-Sainte-Catherine Road, Montreal, Quebec H3T 1E2 Canada; 2 Department of Family Medicine, McGill University, 5858 Côte-des-Neiges Road, 3rd floor, Montreal, Quebec H3S 1Z1 Canada; 3 Desautels Faculty of Management, McGill University, 1001 Sherbrooke Street West, Montreal, Quebec H3A 1G5 Canada

**Keywords:** Mixed method, Primary care, Alzheimer’s disease and related dementia, knowledge translation

## Abstract

**Background:**

Passive dissemination of information in healthcare refers to the publication or mailing of newly established guidelines or recommendations. It is one of the least costly knowledge translation activities. This approach is generally considered to be ineffective or to result in only small changes in practice. Recent research, however, suggests that passive dissemination could, under certain conditions, result in modifications of practice, similar to more active dissemination approaches. The objective of our study was to uncover the conditions associated with the change in primary care practice, namely Family Medicine Groups (FMGs) in Quebec (Canada), following the passive dissemination of recommendations for the diagnosis and management of Alzheimer’s disease and related dementia (AD).

**Methods:**

We used a three-step, innovative, convergent mixed methods design based on a multiple case study in eight FMGs. Two studies were conducted in parallel: (1) a before and after retrospective chart review and a cluster analysis of FMGs performed on two clinical performance indicators—the rate of AD diagnosis and the quality of follow-up care; (2) a qualitative descriptive study using interviews and focus groups with FMG clinicians and healthcare managers. The results were integrated using joint displays.

**Results:**

After the passive dissemination of the recommendations, some FMGs started to implement the recommendations while other FMGs did not change their practice with respect to the AD diagnosis rate and quality of follow-up care. Three interrelated conditions were identified for the successful passive dissemination of clinical recommendations: (1) FMG clinicians with a moderate to high baseline expertise and confidence, which was linked to their existing collaboration with hospital-based specialists in dementia and their motivation; (2) the presence of a self-identified champion (individual champion or collective championship) in the FMGs taking the lead, motivating the clinical staff or organizing training; (3) the availability of sufficient clinical staff enabled these two conditions to have an impact on the implementation of recommendations through passive dissemination.

**Conclusions:**

Passive dissemination of clinical recommendations, a low-cost knowledge translation approach, may lead to practice change under some specific conditions. More active dissemination efforts may only be needed in sites where these conditions are absent.

**Electronic supplementary material:**

The online version of this article (10.1186/s13012-018-0822-x) contains supplementary material, which is available to authorized users.

## Background

Knowledge translation (KT) in healthcare research refers to “the incorporation of research findings into health policy and routine clinical practice” [1]. It is divided into passive and active dissemination strategies [2, 3]. Passive dissemination (just let it happen) [4] involves untargeted publication or mass mailing of information [5–7]. It implies no tailoring of the message and no planning or control of the delivery and is one of the least costly KT activities [5, 8, 9]. In contrast, active dissemination (make it happen) [4] implies an active process of information communication including targeting and packaging the information for end users [7, 10]. In this case, the message is tailored for particular end users, and its implication for a specific practice is emphasized [10]. It commonly uses costly organizational and behavioral tools such as continuing education, outreach, audit/feedback, and decision support system [3, 10–12]. Active dissemination is generally thought to be an effective form of communication to achieve change in clinical practice [10].

Contrary to active dissemination, passive dissemination of clinical guidelines or recommendations is generally considered to be ineffective or to result in small clinical practice changes [10, 12–14]. End users of passively disseminated messages tend to be already open to the message and actively seeking the information, limiting message penetration [7, 10]. The use of passive dissemination has declined over the past decade [9]; however, the debate on its benefits and impact has recently been reopened. A review concluded that passive strategies could have an impact on practice at a lower cost and with a higher feasibility than active dissemination strategies [5]. Knapp et al. [15] found similar levels of guideline implementation in clinical practice for asthma and croup using both active and passive dissemination approaches. A recent RCT found a very limited additional impact from active strategies in stroke [16]. Under specific conditions, passive dissemination of guidelines or recommendations could thus be as effective in modifying clinical practice as more active dissemination approaches. However, little is known about the conditions that are specifically linked to an impact of passive dissemination.

Many conditions linked to effective KT strategies have already been studied, in particular, the characteristics of the desired change and the characteristics of the knowledge users [5]. Contextual factors have been identified including organizational support, culture/climate of the organization, adequate financial and human resources, topic/issue as a priority, local champion, degree of formalization of tasks, and centralization of power [12, 17–19]. Yet, evidence on contextual factors still remains scarce [4, 5] and did not differentiate between the type of KT strategy used (passive versus active dissemination).

The objective of our study was to uncover the contextual conditions associated with the change in primary healthcare practice following the passive dissemination of recommendations for older patients with Alzheimer’s disease and related dementia (AD) in Quebec, Canada.

## Methods

### Context

In May 2009, an expert report on the diagnosis and management of AD was released to the Quebec Ministry of Health (Canada) [20]. The Ministry of Health used a passive dissemination strategy and published the recommendations from the report on their website and held a press conference for the general public (see Additional file 1 for more details). Certain interdisciplinary primary healthcare organizations, known as Family Medicine Groups (FMGs), implemented the recommendations, while others did not. This passive dissemination of recommendations offers an excellent opportunity to identify organizational conditions linked to practice change.

### Study design

We used an innovative convergent mixed methods design based on a multiple case study approach, which is recognized as an ideal method to study organizational conditions and impact of passive KT strategies [4].

A quantitative before-after retrospective chart review and cluster analysis was conducted [21] as well as a qualitative descriptive study. Both sets of data were compared and integrated.

### Site selection

We used a purposeful maximum variation sampling strategy [22] based on region (metropolitan/semi-rural), date of creation, and size (number of registered patients/clinicians). Accordingly, we selected eight FMGs, which were considered a sufficient sample for a cluster analysis with two variables [23]. Table 1 shows the characteristics of the sites.

**Table 1:** Characteristics of the Family Medicine Groups

Site	Region	Date of creation	Study period (chart review)	Date of interviews/focus groups	Number of registered patients	Number of family physicians	Number of nurses	Number of participants to interview and focus groups				
								MD	RN	MAN	SPE	Total
A	Metropolitan	2003	Oct. 2008–Jul. 2009 (PRE) Oct. 2011–Jul. 2012 (POST)	2012/2014	19,000	12	9	6	2	1	0	9
B	Semi-rural	2005	Oct. 2008–Jul. 2009 (PRE) Oct. 2011–Jul. 2012 (POST)	2012/2014	10,800	7	3	6	3	0	0	9
C	Metropolitan	2008	Oct. 2008–Jul. 2009 (PRE) Oct. 2011–Jul. 2012 (POST)	2012/2014	17,500	28	2	2	2	2	2	8
D	Semi-rural	2009	Oct. 2009–Jul. 2010 (PRE) Oct. 2012–Jul. 2013 (POST)	2013/2015	7000	7	2	6	2	1	0	9
E	Semi-rural	2009	Oct. 2009–Jul. 2010 (PRE) Oct. 2012–Jul. 2013 (POST)	2013/2015	4000	2	2	2	1	0	0	3
F	Semi-rural	2009	Oct. 2009–Jul. 2010 (PRE) Oct. 2012–Jul. 2013 (POST)	2013/2015	3166	2	2	2	1	0	0	3
G	Metropolitan	2004	Oct. 2008–Jul. 2009 (PRE) Oct. 2011–Jul. 2012 (POST)	2012/2014	15,000	19	3	1	1	0	1	3
H	Semi-rural	2007	Oct. 2008–Jul. 2009 (PRE) Oct. 2011–Jul. 2012 (POST)	2012/2014	3600	8	4	7	4	1	0	12
All	–	–	–	–	80,066	85	24	32	16	5	3	56

*MD* physicians, *RN* nurses, *MAN* healthcare managers, *SPE* specialist physicians

### Quantitative study

A retrospective chart review was performed in two 9-month study periods, before (PRE) and after (POST) the passive dissemination of the recommendations. The PRE and POST study periods were, respectively, chosen based on the date of the site’s creation and the date when the recommendations were passively disseminated to the sites (Table 1).

#### Population

Chart data were collected on two populations of interest: the general population of patients aged 75+ and patients aged 75+ with AD. For the general population, 75 charts were randomly selected per site, per study period. Inclusion criteria were 75+ and with at least one visit to their FMG during the period. For the subgroup of patients with AD, approximately 40 charts were randomly selected per site, per study period.

#### Outcomes

Based on a logic model (Additional file 2), we selected two clinical performance indicators: rate of AD diagnosis and quality of follow-up care. The rate of diagnosis was calculated from the general population as a proportion of patients 75+ with a diagnosis of AD made by the site within the study period. A quality of follow-up care score—combining assessments of cognition, functional status, behavioral and psychological symptoms of AD, caregiver needs, driving, medication, weight, anticholinergic prescriptions, and referral to homecare services and Alzheimer Societies [24]—was derived from ACOVE [25] and the Canadian recommendations on AD [26]. The follow-up score was calculated for the subgroup of patients with AD as the proportion of assessments performed over the total number of applicable assessments [26].

### Qualitative study

We recruited 56 participants (32 family physicians, 16 nurses, 5 healthcare managers, and 3 specialists) (Table 1). We aimed to recruit at least 1 physician and 1 nurse key informant [22] per site and expanded our recruitment process to ensure both within-site and between-site data saturation [27]. During the year following the passive dissemination of the recommendations (2012–2013), 26 individual interviews were conducted by two researchers (IV, LL). The interview guide (Additional file 3) included themes on the structure of the FMG, current interventions and practice for older patients with AD, barriers and facilitators to the adoption and use of the recommendations, and their impact. The interview guide was piloted and refined with four experts from different domains: family medicine, nursing, public health, and geriatrics. To triangulate our data [22], we also conducted a focus group in each site following the chart review (2014–2015). All team members were invited to participate. We presented their site-specific and overall results of the chart review and asked the clinicians to discuss the results to develop a collective understanding of the barriers and facilitators in the implementation of the recommendations. These focus groups included a total of 30 participants and were conducted by four researchers (IV, LL, GAL, LV). Interviews and focus groups lasted 1 h on average and were recorded and transcribed. Field notes were taken during the focus groups and interviews and during informal discussion between the research team and the clinicians. The data collection process resulted in 750 pages of transcripts.

### Analysis and integration of quantitative and qualitative results

The mixed methods analysis comprised a three-step process: (1) descriptive quantitative analysis and cluster analysis, (2) thematic analysis of the qualitative data, and (3) integration of quantitative and qualitative results.

#### Step 1: Descriptive quantitative analysis and cluster analysis

In each site and period, the AD diagnosis rate was calculated over the 9-month period and adjusted to a 1-year rate. Rates were also standardized to the age and sex distribution of the Quebec population [28] to ensure comparability across sites. Line graphs of these adjusted rates in the PRE and POST period of each site were produced.

Cluster analyses were conducted to determine the subgroups of sites with similar profiles in terms of diagnosis rate and quality of follow-up, respectively. Analyses were based on the Ward method, a hierarchical, agglomerative approach which uses minimum variance criteria to determine site grouping [29]. For each indicator, two variables were submitted to the cluster analysis: the PRE value and the change value between the PRE and POST periods. The number of clusters chosen was based on the graphical display of the clusters (dendrograms).

#### Step 2: Thematic inductive analysis

Thematic inductive analyses [30] using QSR N’Vivo 10 (QSR International Pty Ltd. Version 10, 2012) of the data collected from the interviews, focus groups, and field notes were performed to understand the contextual conditions, barriers, and facilitators to the implementation of recommendations. We proceeded with a round of open line by line coding by rephrasing and summarizing the content of the verbatim. We then grouped the codes into categories (see the list of categories presented in Additional file 4). Finally, we regrouped the categories into different levels: patient, site (intra-organizational), network (inter-organizational), and system levels. The analyses were conducted by three researchers in parallel (IV, LL, MLB) for the first 10% of the verbatim, and the remaining verbatim was coded by one researcher (IV) and validated by another (MLB). The participants’ recruitment and the analysis process were iterative until a consensus was reached among coders. Data collected through interviews and focus groups were merged since the first round of analyses did not reveal substantive information when the data sources were considered independently. We did, however, keep track of the data source even after merging.

#### Step 3: Integration of qualitative and quantitative results

Finally, the results of the cluster analysis were integrated with the results of the thematic analysis using joint displays [31]. Joint display brings qualitative and quantitative data together through a visual means to “draw out new insights beyond the information gained from the separate quantitative and qualitative results” [31]. We thus developed a table where columns represented clusters and rows represented qualitative themes in order to identify the contextual conditions that differentiated the clusters. Common factors across clusters, such as system-level barriers, were not retained as they could not explain the differences between the clusters.

## Results

### Descriptive and cluster analysis (step 1)

**Fig. 1:**
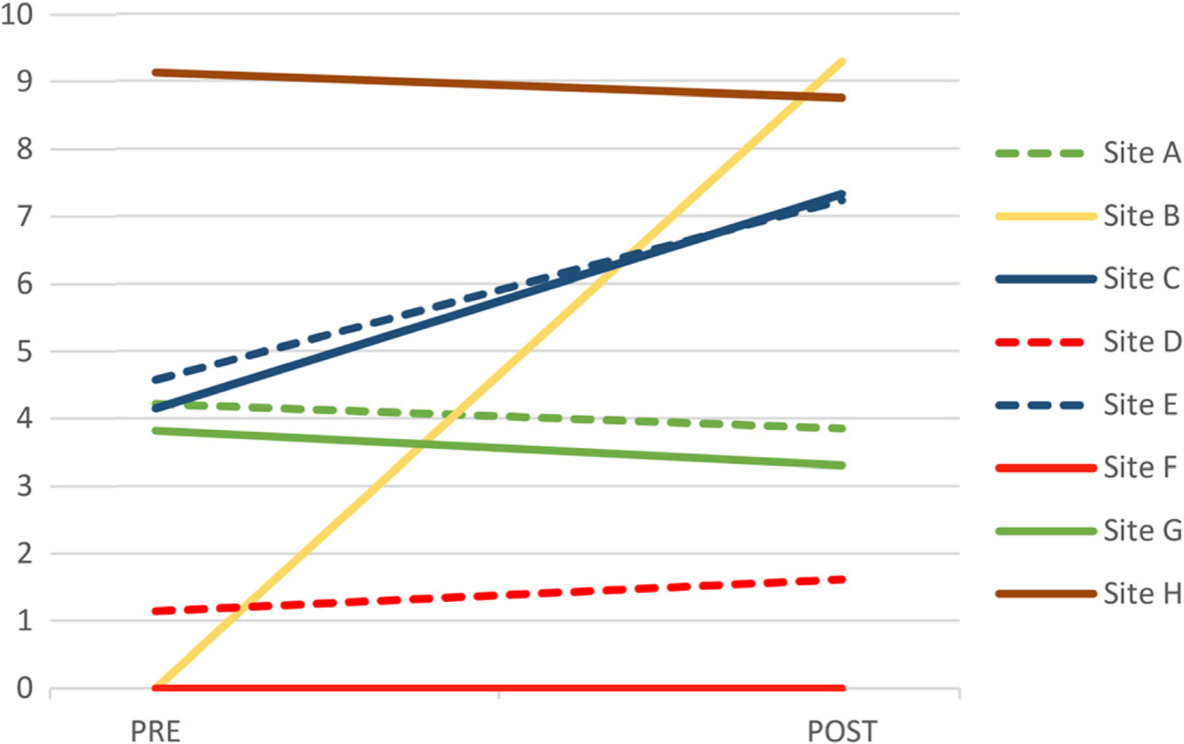
Alzheimer’s disease and related disorders (AD) diagnosis rate by Family Medicine Groups per 100 person-years. Sites from cluster D1 are represented in green, sites from cluster D2 are represented in dark blue, sites from cluster D3 are represented in red, sites from cluster D4 are represented in yellow, and sites from cluster D5 are represented in brown

**Fig. 2:**
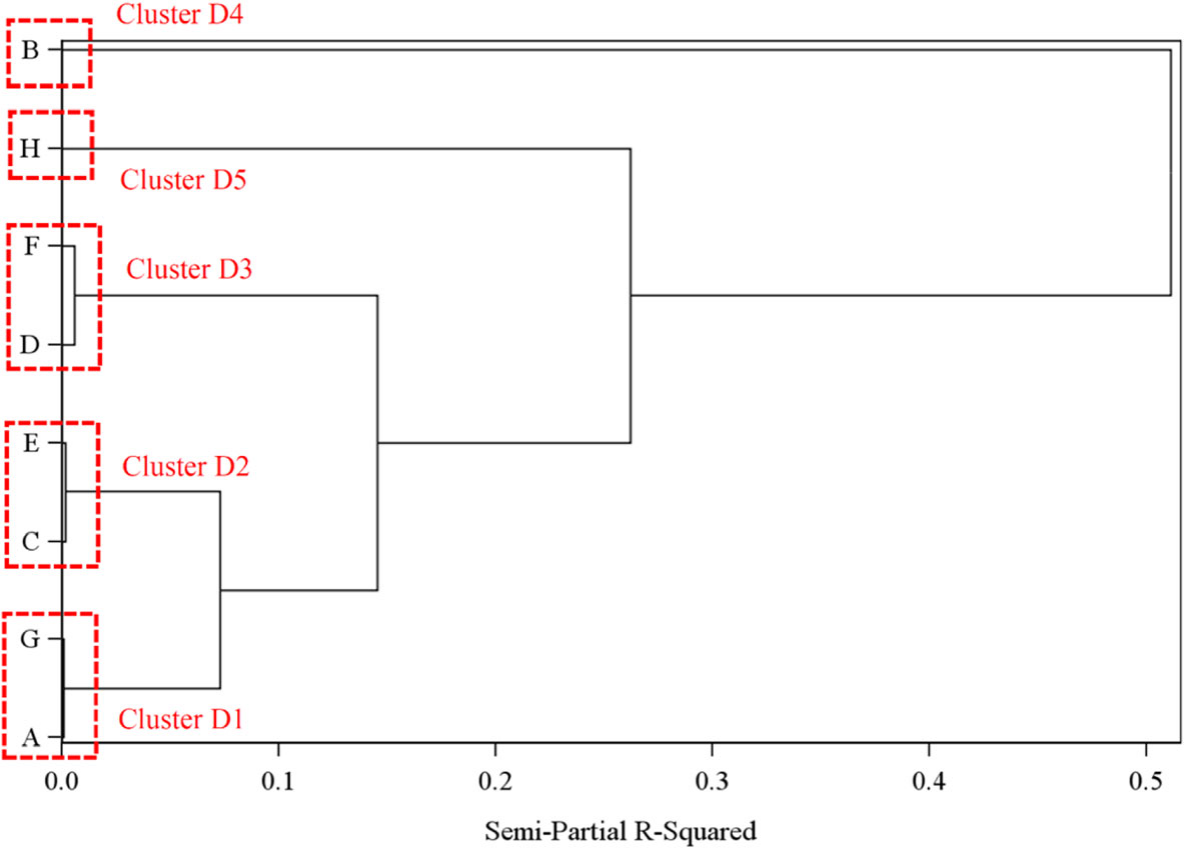
Family Medicine Groups cluster identification related to the diagnosis rate from dendrogram analysis

In terms of the AD diagnosis rate, we observed a range of PRE and POST rates across the sites (Fig. 1). Five clusters of sites were identified: cluster D1 (sites A, G), cluster D2 (C, E), cluster D3 (D, F), cluster D4 (B), and cluster D5 (H) (Fig. 2). As seen in Fig. 1, sites A and G started with a moderate PRE diagnosis rate that remained stable in the POST period. Sites C and E started with a moderate PRE diagnosis rate which increased considerably in the POST period. Sites D and F started with a low PRE diagnosis rate that remained stable. Site B started with a low PRE diagnosis rate which increased considerably in the POST period. Site H started with a high PRE diagnosis rate that remained stable in the POST period.

**Fig. 3:**
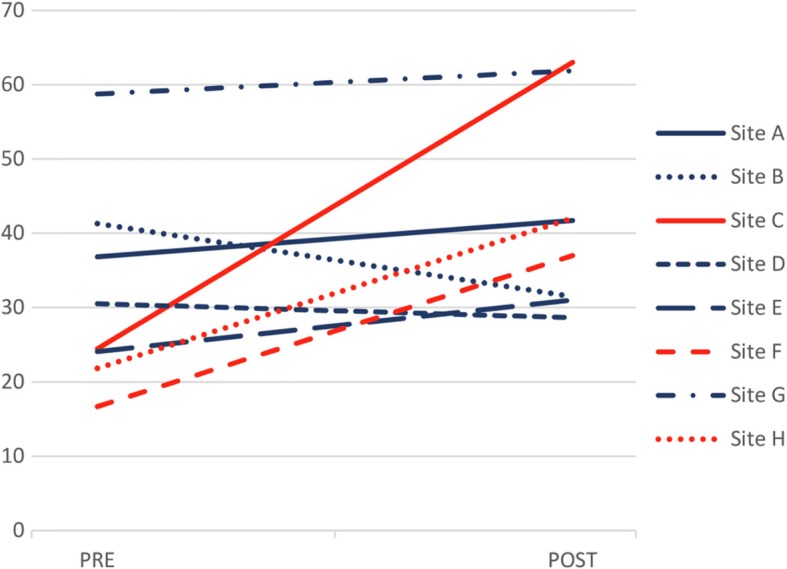
Quality of follow-up by Family Medicine Groups of patients with Alzheimer’s disease and related neurocognitive disorders (AD) (score in percentage of items). Sites from cluster F1 are represented in red, and sites from cluster F2 are represented in dark blue

**Fig. 4:**
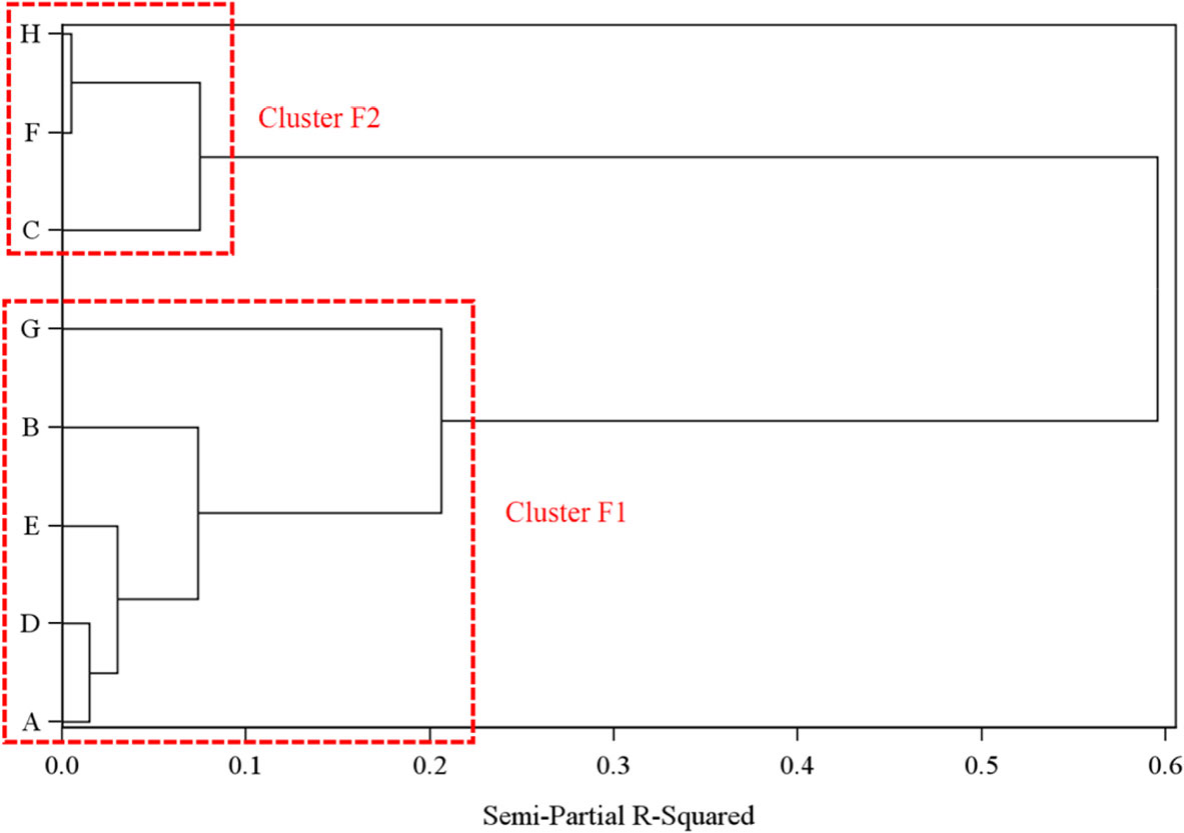
Family Medicine Groups cluster identification related to the quality of follow-up care from dendrogram analysis

In terms of the quality of follow-up scores, we also observed a variety of PRE and POST scores across the sites (Fig. 3). Two clusters of sites were identified: cluster F1 (sites A, B, D, E, G) and cluster F2 (C, F, H) (Fig. 4). As seen in Fig. 3, Sites A, B, D, E, and G all started with a high PRE score that remained stable in the POST period. Sites C, F, and H started with a lower PRE score followed by a marked increase in the POST period.

### Thematic inductive analysis (step 2)

A comprehensive list of barriers and facilitators was identified and grouped into four levels: patient, site (intra-organizational), network (inter-organizational), and system levels (see Additional file 4 for the complete list of themes). The key conditions that differentiated the clusters based on the integration of the cluster analysis and the qualitative results are presented in the following paragraphs.

### Integration of qualitative and quantitative results (step 3)

#### Conditions related to changes in AD diagnosis rate following passive dissemination of recommendations

Two key and interrelated conditions were essential: a moderate to high initial level of expertise and confidence with regard to AD and the presence of an FMG self-identified champion (see Additional file 5). For each cluster, we will describe these two conditions, their underlying drivers and the characteristics of the champions.

*Clusters D1* (*maintaining operations*) *and D2* (*going even further*) shared the same initial conditions—a moderate expertise and confidence of a family physician (FP). They identified some difficulties or limitations with making the diagnosis.

Because sometimes, when we’re talking in the office and I know the person well, he’ll be very interactive and social and I’ll miss the fact that their cognitive functioning is impaired. FP, site G (cluster D1)

Unfortunately, the colleague who received the formation went on sick leave so we limited our activities a bit. We tried to prioritize… I don’t like the word but, we tried to focus on what was a priority for the clinic. So maybe we went for chronic diseases with physical impacts such as diabetes, hypertension etc. Unfortunately, cognitive impairments, as far as management goes, have not really received that much attention. We continue to screen for it, we’re trying to do it as much as possible with all the patients we meet and all the patients who need it. FP, site C (cluster D2)

Their expertise and confidence relied mostly on two different drivers. First, these clusters had access to resources, which they felt were useful. They also had developed valuable relationships with experts, from whom they could learn and receive training or guidance.

We also have a geriatrician assigned to us who can give us a kind of training/conference. […] On at least two occasions the geriatrician has given us training sessions on dementia-related subjects. FP, site G (cluster D1)

We have a typical referral process in place. They […] typically provide a very good note back to us, better than most other specialists who sometimes never even send anything back. It’s too common that we don’t get back any letters from consultants considering that communications means are really suboptimal. I think with the memory clinics, it’s much better. FP, site A (cluster D1)

If I need to, it’s easy to make referrals and collaborate. Also, it’s easy to make referrals to psychogeriatric services when we see behavioral or atypical problems. FP, site E (cluster D2)

Second, both clusters expressed a clear interest in AD, showing their motivation with AD care.

Our medical team is basically high quality. Many of them are always very up-to-date in many different areas. Initially there was a keen interest in Alzheimer disease. FP, site C (cluster D2)

However, while the diagnosis rate did not increase over time for D1, a significant evolution was observed in D2. Unlike cluster D1, cluster D2 had a clear self-identified champion who motivated the clinical staff, organized training, and became a real change agent. The champion recognized the potential benefits of the new recommendations, convinced other colleagues, and provided support and guidance.

Interviewer 1: Do you have a family physician or a healthcare professional who’s assumed a leadership role or an influential role in disseminating information of the [recommendations]?

Respondent: Not to my knowledge. FP, site A (cluster D1)

A physician who had taken an interest worked on maintaining everyone’s determination by regularly shaking up his troops. […] The nurse came with this project and with Dr. X, they motivated the whole medical staff. FP, site C (cluster D2)

*Cluster D3 (no blooming in a dry land)* was characterized by sites that had low initial expertise and confidence with regard to AD and little change in diagnosis rate following the passive dissemination. In this cluster again, the expertise and confidence relied mostly on the two drivers First, the sites from cluster D3 described AD as a low priority, showing their low motivation with AD care.

Interviewer: Can you think of some potential barriers there could be to the implementation of these [recommendations]?

Respondents: The biggest barrier is making dementia one of our objectives, to get people interested in it. There is no doubt about that. FP, site D (cluster D3)

Dementia is a real pain, it’s a lot to absorb. […] And the physician alone, with only two nurses for twelve physicians, we won’t be able to make it. FP, site D (cluster D3)

I’m not comfortable making the diagnosis. I feel insecure. I don’t know how to do it. I’d even say that I’m useless. FP, site F (cluster D3)

Second, FPs were also isolated from major hospitals and did not benefit from service corridors or regular contact with hospital-based specialists in AD (e.g., memory clinics).

They mentioned a lack of training. They can’t go to the meetings because the two clinics are so far apart, the travel time is too great. Dr. X used to go to their clinic occasionally, but he hasn’t been there in a long time. Field notes, site F (cluster D3)

Diagnosis rate did not increase over time as no FP took an active primary role as a champion to disseminate the recommendations.

There used to be a doctor from another FMG who came to see us to talk about dementia, but he doesn’t come anymore. Now there’s no-one in charge of this issue. Focus group, site F (cluster D3)

*Cluster D4 (a particular issue—self-referral)* consisted of a special case. This site had a high initial expertise and confidence. In this cluster again, the expertise and confidence relied mostly on two drivers: access to resources and expert guidance and the motivation with AD care.

I don’t think there was a lack of interest on anyone’s part (…) Everyone is open to the elderly. FP, site B (cluster D4)

It was my luck that geriatricians came to see us at hospital X […] If I need to hospitalize someone, I’ll call the geriatrician who’s on call and it’ll get done, for sure, since I know them and they know me. FP, site B (cluster D4)

Yeah, of course I’m the same person in primary or secondary care […] The nurses here contact my nurse at hospital X and they call each other on a regular basis. FP, site B (cluster D4)

One FP who practiced both in the FMG and in a memory clinic played the role of a champion.

I’ll tell Dr. X [the site’s champion], ‘You should take a look at such-and-such patient…’ That may help a bit, at least it’s reassuring. He’ll tell me, ‘Start with that, give him this med.’ A brief, 5-minute consultation leaning on a counter. It’s having a doctor who’s always on the cutting edge. FP, site B (cluster D4)

However, the initial diagnosis rate was extremely low. This is explained by the fact that this champion FP was self-referring FMG patients to his own hospital-based memory clinic. After the passive dissemination of the recommendations, he decided to move his practice from the hospital to the FMG and diagnose patients within the FMG (informal contact with FP champion from site B) which increased the diagnosis rate from very low to very high. He trained the FMG nurses to support him.

Respondent: Half my career is about Alzheimer’s disease. I already had this project in mind and I was thinking about how to involve the nurses. […]

Interviewer: Did you initiate the project?[…]

Respondent: Yes, I had this in mind since 2004… But I knew it was not the time […] Then the moment came. I talked about it – I had to talk about it – because there were a lot of nurses hours involved. […] We trained them to do cognitive testing. […] We trained all the nurses of the area! But our nurses were the very first to receive the training. FP, site B (cluster D4)

*Cluster D5 (maintaining operations—full-on mode)* was another special case; it demonstrated a possible ceiling effect in diagnosis rate due to a high initial expertise and confidence of FPs. The expertise and confidence relied mostly on the access to resources and expert guidance. As an FP mentioned while talking about maintaining their high performance before and after the release of the recommendations:

We have gotten so good at this point! I think we’re less and less afraid of starting Aricept. […] We can deal with it! FP, site H (cluster D5)

FPs felt supported in the decision-making process and were connected to hospital AD specialists and services. As an FP mentioned while talking about hospital specialists:

We’re not afraid of making a diagnosis since we know we’ll have support […] We know we have several other professionals around us. We aren’t making the decision on our own. FP, site H (cluster D5)

While FPs perceived they were doing a good job, they did not see the need to improve their practice and no champion emerged.

Interviewer: How would you describe the motivation of your FMG to improve the quality of care for Alzheimer’s patients after 2009?

Respondent: This… In fact, it was like a wave because after that, it tapered off, since the doctors referred many of their patients to me for evaluation, and then for follow-up of the treatment. It means that they were highly motivated at first but then, at some point, it was as if they forgot a bit. Then you would have to go talk about it again. It would need to be discussed regularly if you want them to continue. Nurse, site H (cluster D5)

#### Conditions related to changes in quality of follow-up following passive dissemination of recommendations

Three key and interrelated conditions were essential: a moderate to high initial level of expertise and confidence, the presence of an FMG self-identified champion, and sufficient available professional resources (see Additional file 6). For each cluster, we will describe these three conditions, their underlying drivers, characteristics of the champions, and the available resources.

*Cluster F1 (maintaining operations)* demonstrated a high initial expertise and confidence regarding AD management which relied on two drivers. First, the healthcare professionals reported clinical experience with the population.

Of course, our patients are aging. So, the more we see our patients being affected, the more we are interested in being well-trained. FP, site G (cluster F1)

Second, these professionals had a clear motivation to work with AD, identifying it as one of their priorities.

Interviewer: The implementation of this project, the screening and management of patients with cognitive impairment or AD in your FMG, can you tell us the story of how it went?

Respondent: The story is that it is probably back in 2004, when we became an FMG, we had our first clinical meeting with the nurses in order to prioritize. It was all new for us to work with nurses: which clinical tasks should we ask from them? So, we gave everyone a chance to speak, and we set some priorities. So, the first priority was Coumadin […] After that, it was diabetes, and not long after, Alzheimer disease. FP, site B (cluster F1)

Two different conditions contributed to the lack of improvement in the quality of follow-up. In two sites (A, G), there was no champion to provide specific mentoring and support to the nurses.

Interviewer 1: Was that really handled by just one person?

Respondent: No, no, […] We do not really have one doctor with a specific interest in dementia. FP, site G (cluster F1)

Interviewer: [Did the] organization have a family physician or a healthcare professional who has assumed a leadership role or an influential role in disseminating information of the Bergman report?

Respondent: Not to my knowledge. FP, site A (cluster F1)

In the other three sites (B, D, E), a lack of nurses was reported during the period (due to maternity leaves), and clinical staff was not available to implement the new recommendations.

Interviewer: Oh, when did the nurse leave?

Respondent 1: She retired in October but she went on sick leave in March for the whole winter. […] Maybe this can explain why there was less screening during that period… […] It’s really because Nurse X wasn’t there. We had just one nurse for all the doctors here. Nurse, site D (cluster F1)

Interviewer: So next, looking at the quality of follow-up results. […] We were at 40% in 2008-2009 and we had decreased to 25% by 2011-2012.

Respondent 1: Well, it’s the maternity leave […] there were some substitutes, we had some substitutes, sure, but they could not do it.

Respondent 2: And there were a lot of changes. We had many substitutes during 2008-2009. FP 1 and nurse 1, focus group, site B (cluster F1)

*Cluster F2 (stepping up, learning, and moving forward)* was characterized by low initial expertise and confidence which was explained by two drivers. First, there was a lack of clinical experience due to the small number of older patients in each site. As an FP explained while talking about the type of clientele the FMG served:

It’s the region […] where we find the youngest client group […] there isn’t a geriatric client base around that clinic. FP, site F (cluster F2)

This lack of clinical experience was also a result of the minimal availability of nurses with expertise in AD management.

Before the training with Dr. X, we were wondering a bit about [AD]. I had a nurse working with me […], who was particularly interested, who had gotten some information from Dr. Y […] so [the nurse] had already assembled a minimum of information with the resources at our disposal but [the nurse] went on long-term sick leave. Nurse, site B (cluster F1)

Second, the low initial expertise and confidence was also due to a low support from home-based and community-based services.

The Alzheimer Society never calls back, they don’t provide the required services. […] We can call them, but they don’t follow up. […] The waiting lists are long, particularly for [home-based] services. […] We can do very good work, but we can’t do it on our own. FP, site H (cluster F2)

Despite low initial expertise and confidence, these sites showed significant improvement in the quality of follow-up. The presence of a self-identified champion reaching out to the nurses motivated change within the clinical team. In two of the sites (C, F), the champion was an FP who worked closely with nurses to engage them in implementing the recommendations. In the third site (H), it was a collective championship of nurses very closely connected and involved in the implementation process.

I said: ‘Well I’m ready, ready to carry the torch to all the general practitioners, first in my clinic, and then, to see what I can do in the region. FP, site C (cluster F2)

The FMG nurses, we were trained first. […] We spoke about it to the others, because we had a binder, so that’s why there was an increase. It isn’t just me […]. Yes, we’re the team. Nurses 2, 3, and 4, focus group at site H (cluster F2)

## Discussion

Our results suggest that the passive dissemination of recommendations on the diagnosis and management of AD can result in clinical practice changes in FMGs in terms of two indicators of clinical performance (rate of AD diagnosis and quality of follow-up care). Furthermore, our study is the first to identify the key conditions linked to effective changes in clinical practice following a passive dissemination of recommendations. Three key and interrelated conditions were essential: a moderate to high initial level of expertise and confidence, the presence of an FMG self-identified champion, and sufficient available professional resources (Additional files 5 and 6).

First, our results suggest that a moderate to high initial level of expertise and confidence in the FMG clinical team provided a breeding ground to build on and implement clinical practice changes. The initial level of expertise and confidence of these clusters was linked to the proximity of specialists or AD experts who helped develop the expertise and confidence of the FMG clinicians for AD and their motivation with AD care. Indeed, a clinician who is motivated and has the minimum required skills is more likely to adopt an innovation and change his/her clinical practice [4]. Motivation is also the first step before any change in behavior and adoption of evidence-based practice can occur [32]. This initial expertise and confidence, relying on motivation and access to resources and experts, seemed necessary to identify difficulties or “knowledge-practice gaps” [33], which is the first step for engaging in a process of clinical practice change and adopting recommendations [33]. In the sites where the passive dissemination of recommendations led to a change in clinical practice, the second step (adapting the knowledge to the local context) [33] was undertaken by the sites themselves, as opposed to being imposed by the Ministry of Health or another health authority. The sites had the freedom to adapt the recommendations to their context and develop tools adapted to their practice. These FMGs seized the opportunity and endorsed the recommendations, implementing changes in their clinical practice.

Second, our results suggest that the involvement of a champion was also necessary. The role of a champion is already recognized in the literature as important for practice change [4, 10, 14]. Clinicians rely more on informal social and professional interactions within their communities of practice than on formal published evidence to guide their judgment [34]. From the literature, we know that the role of the champion must be specific in terms of audience, task, and context [35]. In our study, the champions wanted to implement the recommendations for AD diagnosis and management and selected their audience depending on the aspects that needed improvement and tailored the recommendations to the local context and resources on their own, that is, the champions reached out to physicians for the diagnostic rate or to the whole team for the follow-up care, which in turn allowed better AD diagnosis and management. Champions also diffused tacit knowledge by answering questions from colleagues. Tacit knowledge or “know-how” is key in medical and nursing practices [36]. In this study on passive dissemination, champions were self-identified within the FMG practice. Some physicians and nurses within the site decided to play the role of a champion on their own. In this case, the fit and cohesion of champions and their local teams were optimal, which is key to success [37]. Furthermore, in our study, we found that the type of champion varied from one site to another and could either be an individual champion or a collective championship. The collective championship took the form of nurses deciding as a group to take on a championship role. To our knowledge, whereas local individual champions have been extensively described [5, 12, 35, 38], a collective championship has never been identified before. In passive dissemination, there is a bottom-up approach to the engagement of clinicians, which can lead to innovation in the characteristics of champions.

Finally, our results suggest that sufficient available resources are needed. Even if the initial level of motivation and expertise is met and a champion is present, practice cannot change if resources are not sufficient. In the absence of clinical staff or when nurse turnover was high, any champion was unlikely to have much impact. This highlights once again the importance of the availability of sufficient material and human resources [18, 19]. In sum, passive dissemination can work but under specific conditions: all three identified key conditions of a moderate to high initial level of expertise and confidence, the presence of an FMG self-identified champion, and sufficient available professional resources need to be present to ensure the success of practice change.

### Strength and limitations

This study has multiple strengths, starting with its innovative design. In our mixed methods design, both quantitative and qualitative data iteratively strengthened each other and allowed us to disentangle the change process following passive dissemination of clinical recommendations, enriching our understanding of the key conditions linked to their successful implementation [4, 39]. Quantitative data allowed us to objectively measure the practice change. The cluster analysis provided a more objective approach to grouping FMGs than a subjective interpretation of graphs or an arbitrary cutoff value. In addition, qualitative data, with its full richness, allowed us to study the conditions under which better AD diagnosis and management occur.

Our study also presents some limitations. The small number of sites limited the variables that could be included in the cluster analysis. In addition, our study was conducted in only one Canadian province and may well reflect specific contextual aspects. However, the contrasted sampling allowed us to capture a variety of contexts in terms of region, date of creation, and size.

## Conclusion

Given that implementation of clinical recommendations is challenging, a prior understanding of the best conditions for successful uptake is essential [4] in order to customize the implementation strategy and achieve tangible results. Decision-makers and healthcare managers can then use passive dissemination in sites where favorable conditions are present (i.e., in sites with moderate to high initial level of expertise and motivation, self-identified champions, and sufficient resources). Whereas in sites with unfavorable a priori conditions, more active and costly dissemination strategies need to be considered. Based on the results of our study, the Quebec Ministry of Health has further developed the implementation strategy for a Quebec Alzheimer Plan. These results will also be used to implement the federal Alzheimer plan in Canada following the recent passage of Bill-C233, *an Act respecting a national strategy for Alzheimer’s disease and other dementias* [40].

## Additional files

Additional file 1: Recommendations for the diagnosis and management of Alzheimer’s disease and related dementia (AD) in Quebec (Canada). (DOCX 35 kb)

Additional file 2: Logic model. (PDF 181 kb)

Additional file 3: Interview guide. (DOCX 16 kb)

Additional file 4: Results of the thematic analysis (obstacles and facilitators). (DOCX 22 kb)

Additional file 5: Conditions linked to initial diagnosis rate of Family Medicine Groups of clusters and their change. (DOCX 92 kb)

Additional file 6: Conditions linked to initial quality of follow-up care of Family Medicine Groups of clusters and their change. (DOCX 55 kb)

## Abbreviations

AD: Alzheimer’s disease and related dementia
FMG: Family Medicine Group
FP: Family physician
