# Supplementary material for: Shedding light on conditions for the successful passive dissemination of recommendations in primary care: a mixed methods study
Source: Implement Sci. 2018 Oct 16;13:129. doi: 10.1186/s13012-018-0822-x (PMC6192363; doi:10.1186/s13012-018-0822-x)
Supplement: Supplementary file 1 — Recommendations for the diagnosis and management of Alzheimer’s disease and related dementia (AD) in Quebec (Canada). (DOCX 35 kb) [file 13012_2018_822_MOESM1_ESM.docx]

**Additional file 1. Recommendations for the diagnosis and management of Alzheimer’s disease and related dementia (AD) in Quebec (Canada).**

In 2007, the Quebec ministry of health and social services (MSSS) mandated a task force chaired by Dr. Bergman to develop recommendations for a provincial Alzheimer plan. The task force based their recommendation on a patient-centered approach and promotes patients and caregivers’ participation in the care process. They also advocated the integration of health, social and support services - which is based on clinical responsibility and continuity of care that take into account the complexity of the patients’ needs. In 2009, the recommendations was tabled and offered seven priority actions, accompanied with 24 recommendations. The seven priority actions are the following:

1. Raise awareness, inform, and mobilize
2. Provide access to personalized, co-ordinated assessment and treatment services for people with Alzheimer’s and their family/informal caregivers
3. In the advanced stages of Alzheimer’s, promote quality of life and provide access to home-support services and a choice of high-quality alternative living facilities.
4. Promote high-quality, therapeutically appropriate end-of-life care that respects people’s wishes, dignity, and comfort
5. Treat family/informal caregivers as partners who need support
6. Develop and support training programs
7. Mobilize all members of the university, public, and private sectors for an unprecedented research effort

Passive dissemination of the priority action #2: Recommendations for the diagnosis and management of Alzheimer’s disease and related dementia (AD)

On May 6, 2009, the recommendations were released on the MSSS website and a press conference for the general public was organized on May 31, 2009, this was announced on the MSSS website. This press conference focused on priority action #2. From May 2009 to September 2010, the Quebec general press released many news with reference to the recommendations. (see table below for url).

| **Release date** | **Release source** | **url** |
| --- | --- | --- |
| May 6, 2009 | MSSS website | <http://www.publications.msss.gouv.qc.ca/msss/fichiers/2009/09-829-01W.pdf> |
| May 31, 2009 | Quebec general press | <http://www.newswire.ca/fr/news-releases/invitation-aux-medias---marche-de-la-memoire-rona---les-ministres-yves-bolduc-lise-theriault-et-marguerite-blais-presents-pour-recevoir-le-rapport-bergman-537781331.html> |
| May 31, 2009 | MSSS website | <http://publications.msss.gouv.qc.ca/msss/document-000869/> |
| May 31, 2009 | Quebec general press | <https://ici.radio-canada.ca/nouvelle/437084/alzheimer-rapport-marche> |
| January 9, 2010 | Quebec general press | <https://www.ledevoir.com/societe/sante/280753/proteger-son-cerveau> |
| January 6, 2010 | Quebec general press | <http://sante.canoe.ca/news/chealth/4855?newssource=0> |
| September 22, 2010 | Quebec general press | <http://www.pressreader.com/canada/le-journal-de-montreal/20100922/283463547641562> |
| September 22, 2010 | Quebec general press | <https://www.newswire.ca/fr/news-releases/la-societe-alzheimer--presse-le-gouvernement-dagir-et-lance-un-appel-auxelus----nous-ne-pouvons-plus-attendre---546004742.html> |
