# Supplementary material for: Shedding light on conditions for the successful passive dissemination of recommendations in primary care: a mixed methods study
Source: Implement Sci. 2018 Oct 16;13:129. doi: 10.1186/s13012-018-0822-x (PMC6192363; doi:10.1186/s13012-018-0822-x)
Supplement: Supplementary file 2 — Logic model. (PDF 181 kb) [file 13012_2018_822_MOESM2_ESM.pdf]

<table>
<tr><th rowspan="2">Key components</th><th rowspan="2">Reinforce Family Medicine Group (FMG) capacity and competency</th><th colspan="3">Management of patients living with dementia in FMG
Collaboration family physician-case manager</th><th rowspan="2">Pertinent referrals to memory clinics, BPSD team, homecare services, caregivers support</th></tr>
<tr><th>Identification of patients</th><th>Treatment</th><th>Follow-up</th></tr>
<tr><td>Bergman report's recommendations</td><td>Training in FMG

Additional resources and role definition of case manager (pivot nurse) – resources utilisation

Adapted clinical tools and practice guides to the FMG context</td><td>Identification and evaluation of cognitive disorders by family physician or case manager

Dementia diagnosis

Explanation of diagnosis to patient and caregiver</td><td>Prescription of dementia medications when appropriate

Evaluation of patients and caregivers needs

Education and implication of patients and caregivers</td><td>Proactive follow-up of patients and caregivers

Easy access to a clinician for patients and caregiver

Regular assessments by the family physician or case manager of cognition, functional status, behavioral and psychological symptoms of dementia (BPSD), driving, dementia medication, weight, and homecare services and community services' needs

No anticholinergic prescriptions

Assessment of caregiver needs

Coordination of services by case manager</td><td>Agreement between FMG and other services

Collaboration between all health services

Use of criteria for referrals

Shared Clinical Information System

Shared information

Homecare services – caregiver support: rapid referrals of patients and sufficient resources

Memory clinic – BPSD team: appropriate referrals and evaluation within 3 months (1 month for BPSD team)

Access to laboratory tests, CT scans within 1 month

Improve transition between home and long term care</td></tr>
<tr><td>Outputs</td><td>Not evaluated
• Training received
• Knowledge, attitude and practice of FMG clinicians
• Elaboration and systematic use of practice guides and clinical tools in FMG
• Allocated time by additional resources in FMG versus other populations</td><td>• Number of diagnosed patients and type of dementia or cognitive impairment

Not evaluated
• Number of identified patients
• Number of patients evaluated
• Patients and caregivers' education and information</td><td>Not evaluated:
• Number of patients for whom the FMG initiated the treatment
• Evaluation of patients' needs
• Experience of patients and caregivers</td><td>• Quality of Follow-up

Not evaluated
• Number of contacts or visits between patients or their family and the family physician or case manager
• Role of the case manage in care coordination</td><td>Not evaluated:
• Number of referrals, motives and content
• Time between referral and evaluation by homecare services
• Time between referral and evaluation by memory clinic (<3 months)
• Time between referral and evaluation by BPSD team (<1 month)
• Report sent back to FMG
• Number of patients admitted to long term care</td></tr>
<tr><td>Short term results</td><td colspan="5">Improve identification and diagnosis of patient with cognitive impairment by the FMG
Improve the quality of the management by FMG
Not evaluated:
Improve knowledge, attitude and practice of all FMG clinicians
Decrease delays for memory clinic, BPSD teams, home-based services, CT scans
Decrease non pertinent referrals and increase pertinent referrals to memory clinic
Increase clinicians' satisfaction
Increase patients and caregivers' satisfaction and experience
Unexpected results: overcrowding of home-based services and memory clinic; decrease access to programs for other diseases</td></tr>
<tr><td>Long term results</td><td colspan="5">Not evaluated:
Maintain patients' functional status and quality of life
Decrease burden and improve the quality of life of caregivers
Decrease inappropriate service uses and costs
Maintain patients at home</td></tr>
</table>
