# Supplementary material for: Shedding light on conditions for the successful passive dissemination of recommendations in primary care: a mixed methods study
Source: Implement Sci. 2018 Oct 16;13:129. doi: 10.1186/s13012-018-0822-x (PMC6192363; doi:10.1186/s13012-018-0822-x)
Supplement: Supplementary file 3 — Interview guide. (DOCX 16 kb) [file 13012_2018_822_MOESM3_ESM.docx]

**Additional file 3. Interview Guide.**

**Interventions – General questions on your experience**

**How long have you been operating as an FMG?**

**What types of professionals participate in the FMG?**

**What are the clinical priorities of your FMG?**

**What patient management processes have recently been implemented in your FMG, and what tools have been used with people presenting with Alzheimer disease or dementia?**

**Using the expert panel report for the development of an Alzheimer’s disease action plan (the Bergman report), we have selected the recommendations intended more specifically for FMGs. We will read each of the recommendations and ask that you help us understand what you think of it and how you operate in your FMG:**

- **A structured path for patients:**

Implementing a process for identifying people in need of a cognitive assessment

Performing a cognitive assessment and assessing other needs

Arriving at a diagnosis

Explaining the diagnosis to the patient

Developing a service plan

Supporting family caregivers

Re-assessing needs and following patients

- **Use of information technologies**
- **The functioning of the FMG’s team (nurse, family physician, other health professionals)**
- **The FMG’s relations with the other partners**
- **Training on cognitive impairments and dementia**
- **Changes to funding methods**

**Implementation strategy – barriers – facilitators**

**Can you tell me how you have implemented the recommendations of the report of the expert panel?**

- **How were you told that an intervention would be implemented in your FMG?**
- **Who played key roles in the implementation?**
- **How were you prepared for implementation of the interventions?**
- **What were the key success factors for implementing interventions in your FMG?**

**PERCEIVED IMPACTS**

**What has improved or worsened in your identification and management of people with dementia since the implementation of the intervention(s)?**

**We would like to hear your opinion of the impacts (positive or negative) of these interventions on:** (It is important to go through the whole list.)

- Your personal practice

- The other professionals

- Interprofessional collaboration (communications, decision making, coordination of the work, sharing of tasks and responsibilities)

- Your collaboration with resources outside your organization

- Service utilization and health care costs

- Quality of care

- The application of clinical guidelines

- The patients (health status, quality of life, self-care)

- Their family caregivers
