# Supplementary material for: Shedding light on conditions for the successful passive dissemination of recommendations in primary care: a mixed methods study
Source: Implement Sci. 2018 Oct 16;13:129. doi: 10.1186/s13012-018-0822-x (PMC6192363; doi:10.1186/s13012-018-0822-x)
Supplement: Supplementary file 4 — Results of the thematic analysis (obstacles and facilitators). (DOCX 22 kb) [file 13012_2018_822_MOESM4_ESM.docx]

**Additional file 4. Results of the thematic analysis (obstacles and facilitators).**

| **Barriers at the patient level** | **Facilitators at the patient level** |
| --- | --- |
| Complexity of patient with Alzheimer disease or related dementia (AD) and their caregiver needs   - AD patients are really demanding: they need complex care: medical, nursing, psycho-social interventions - FMGs clinicians feel overwhelmed sometimes and prefer sending patients to specialists - Difficulty in managing the patient and his/her caregiver at the same time - Family physicians do not feel comfortable with the stigma and consequences associated with AD (e.g. driving cessation) - Multimorbidity: family physicians tend to focus on urgent and physical problems rather than chronic and cognitive problems | Interesting role of FMGs clinicians towards older patients with AD   - Rewards from patients and their families - Comprehensive care: care of the family, psychosocial aspects of patients with AD |
| **Barriers at the Family Medicine Group (FMG) level – intra-organization** | **Facilitators at the FMG level – intra-organization** |
| Lack of knowledge, competency   - Lack of family physicians/nurse expertise - Difficulty to differentiate between screening and case finding; normal aging and the symptoms of disease - Lack of knowledge about medication, how to assess driving ability, how to disclose diagnosis, how to support family caregivers   Conflicting priorities and limited resources   - FMG shoulders many priorities that are difficult to manage at the same time : new chronic disease management interventions for diabetes, heart failure, chronic obstructive pulmonary disease, etc.; priority given to orphan patients - Inappropriate ratio of family physicians / nurses   Lack of availability of FMGs nurses   - Turn-over of nurses - Time spent on other populations (diabetes, cardiovascular disease, heart failure)   Limited role of nurses within FMG   - Nurses considered by some family physicians as executants rather than collaborators | Presence of self-identified champion(s)   - Individual champion or collective championship: Champions (family physicians, nurses) play the role of change agents, convince peers, are mentors, are role models - Logistical support to champions: program manager, administrative support   Expertise in AD   - Moderate to high baseline expertise of the FMGs clinicians and their motivation to change - Presence of Family physicians and nurses who have expertise in AD and who are available: who can respond to questions from other clinicians - Recent training in AD   AD is an endorsed priority by the FMG   - Motivation of Family physicians and nurses - Tools have been developed to help clinicians manage their AD patients (algorithms) - Role of nurses and Family physicians and other healthcare professionals (e.g. social workers) have been collectively discussed   Availability of clinical staff   - Availability of FMGs nurses - Nurses play a key role in managing patients with AD - Family physicians work closely with nurses to diagnose and manage AD patient - Extended role of nurses : they perform tests and also play the role of case manager - navigator |
| **Barriers at the network level – inter-organization** | **Facilitators at the network level – inter-organization** |
| Lack of clarity on the role of the different services   - Lack of clarity about the available services and their specific role: memory clinics, teams specialized in behavioral and psychological symptoms of dementia, home-based services - Lack of clarity about the indications to refer a patient to specialized services   FMG lack of access to specific services   - Lack of timely access to specialists - Lack of availability of tests (structural imaging). Family physicians are tempted to send the patient to a memory clinic (or a specialist in cognition) to get easier access to brain imaging.   Lack of information exchange and collaboration between FMG and Emergency Departments (ED), hospitals   - Information regarding a patient visit to ED not available, report from ED after a patient visit sent long afterward or not at all - Report written without precise indication on the action that should be taken by FMG   Lack of information exchange between FMG and home-care services   - No information on what is done by home-care services after the referral, when the assessment of needs occurred, what services are planned etc. | Close collaboration between FMG and hospital-based specialist   - Existence of a Family physician within the FMG who also works in a memory clinic - Local specialist leader: specialist who believes in the ability of FMG to diagnose, treat and follow-up AD patients - Availability of specialists - Mentoring from specialists. E.g. supervision when prescribing an AD medication for the first time, possibility to discuss more complex cases - Link between the FMG nurses and the nurses in specialized clinics: mentoring for the FMG nurses - FMG: positive experience with shared care with specialists if the patients’ conditions are complex   Close collaboration between FMG and other services (home care, community-based services)   - Exchange of information on the care plan developed by home-care services   Developed network between FMGs   - Communities of practice, shared tools |
| **Barriers at the system level** | **Facilitators at the system level** |
| Lack of appropriate guidelines adapted to primary healthcare   - Lack of clinical guidelines integrating AD care with the care of other chronic diseases   Lack of incentive or adverse incentives   - Absence of incentive for home-care services to admit patients directly from FMG. But incentive for home-based services to help in freeing hospital beds and ED - Bureaucratization of some aspects of care   - Forms for exception drugs. Some Family physicians are tempted to send patients to specialists to complete the forms.   - Considerable paperwork to refer a patient to home-based services - Absence of an economic model for the interventions of a visiting specialist - Remuneration – fee for service | Reform of primary healthcare   - Group practice: Family physicians working together with nurses (or other healthcare professional)   Policy direction for AD   - Bottom-up approach based on innovation in the field and best practices - Recommendations are adapted to FMGs |

Notes: AD: Alzheimer Disease or Related Dementia; FMG: Family Medicine Group; ED: Emergency Departments
