# Supplementary material for: Shedding light on conditions for the successful passive dissemination of recommendations in primary care: a mixed methods study
Source: Implement Sci. 2018 Oct 16;13:129. doi: 10.1186/s13012-018-0822-x (PMC6192363; doi:10.1186/s13012-018-0822-x)
Supplement: Supplementary file 5 — Conditions linked to initial diagnosis rate of Family Medicine Groups of clusters and their change. (DOCX 92 kb) [file 13012_2018_822_MOESM5_ESM.docx]

**Additional file 5: Conditions linked to initial diagnosis rate of Family Medicine Groups of clusters and their change.**

A. Cluster D1

Cluster D1

Moderate level of expertise and confidence

AND

Lack of champion targeting the physicians

Moderate initial level

No evolution

B. Cluster D2

Cluster D2

Involvement of a champion targeting the physicians

AND

Moderate level of expertise and confidence

Moderate initial level

Evolution

C. Cluster D3

Cluster D3

Low initial level of expertise and confidence

Lower initial level

AND

Lack of champion targeting the physicians

No evolution

D. Cluster D4

High initial level of expertise and confidence

Cluster D4

AND

Involvement of a champion targeting the physicians

AND

High self-referencing pattern

Evolution

Lower initial level

E. Cluster D5

Cluster D5

Very high initial level of expertise and confidence

AND

Lack of champion targeting the physicians

No evolution

High initial level
