# Supplementary material for: Shedding light on conditions for the successful passive dissemination of recommendations in primary care: a mixed methods study
Source: Implement Sci. 2018 Oct 16;13:129. doi: 10.1186/s13012-018-0822-x (PMC6192363; doi:10.1186/s13012-018-0822-x)
Supplement: Supplementary file 6 — Conditions linked to initial quality of follow-up care of Family Medicine Groups of clusters and their change. (DOCX 55 kb) [file 13012_2018_822_MOESM6_ESM.docx]

**Additional file 6: Conditions linked to initial quality of follow-up care of Family Medicine Groups of clusters and their change.**

A. Cluster F1

Lack of personnel

Cluster F1

OR

AND

High initial level of expertise and confidence

Lack of champion targeting the nurses

No evolution

High initial level

B. Cluster F2

Cluster F2

Involvement of a champion targeting the nurses

Available personnel

Low initial level of expertise and confidence

OR

AND

AND

Presence of a collective

championship

Lower initial level

Evolution
